# Two additional males with X‐linked, syndromic mental retardation carry de novo mutations in *HNRNPH2*

**DOI:** 10.1111/cge.13580

**Published:** 2019-06-24

**Authors:** Wayne M. Jepsen, Keri Ramsey, Szabolcs Szelinger, Lorida Llaci, Chris Balak, Newell Belnap, Cherae Bilagody, Matthew De Both, Raj Gupta, Marcus Naymik, Richa Pandey, Ignazio S. Piras, Meredith Sanchez‐Castillo, Sampathkumar Rangasamy, Vinodh Narayanan, Matthew J. Huentelman

**Affiliations:** ^1^ Translational Genomics Research Institute (TGen) Phoenix Arizona; ^2^ TGen's Center for Rare Childhood Disorders (C4RCD) Phoenix Arizona; ^3^ School of Life Sciences Arizona State University Tempe Arizona

## Abstract

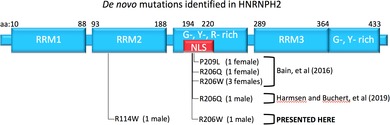

1

*To the Editor*:

This letter is intended to directly respond to and complement that submitted by Harmsen et al in which they describe a male with mental retardation, X‐linked, syndromic, Bain‐type (MRXSB) due to a de novo hemizygous mutation in *HNRNPH2* (c.617G>A, p.Arg206Gln)^1^ previously identified in females by Bain et al and presumed to be embryonically lethal in males.^2^

We have identified two additional males with similar phenotypes carrying de novo mutations in *HNRNPH2*. Patient A is hemizygous for a second MRXSB mutation originally identified by Bain et al in three females within the nuclear localization sequence of *HNRNPH2* (c.616C>T, p.Arg206Trp),^2^ serving as conclusive evidence that known MRXSB mutations are not embryonically lethal in males. Patient B is hemizygous for a private mutation in the second RNA recognition motif (RRM2) of *HNRNPH2* (c.340C>T, p.Arg114Trp), suggesting that other mutations within this gene are capable of producing a range of similar phenotypes (Figure 1).

**Figure 1:**
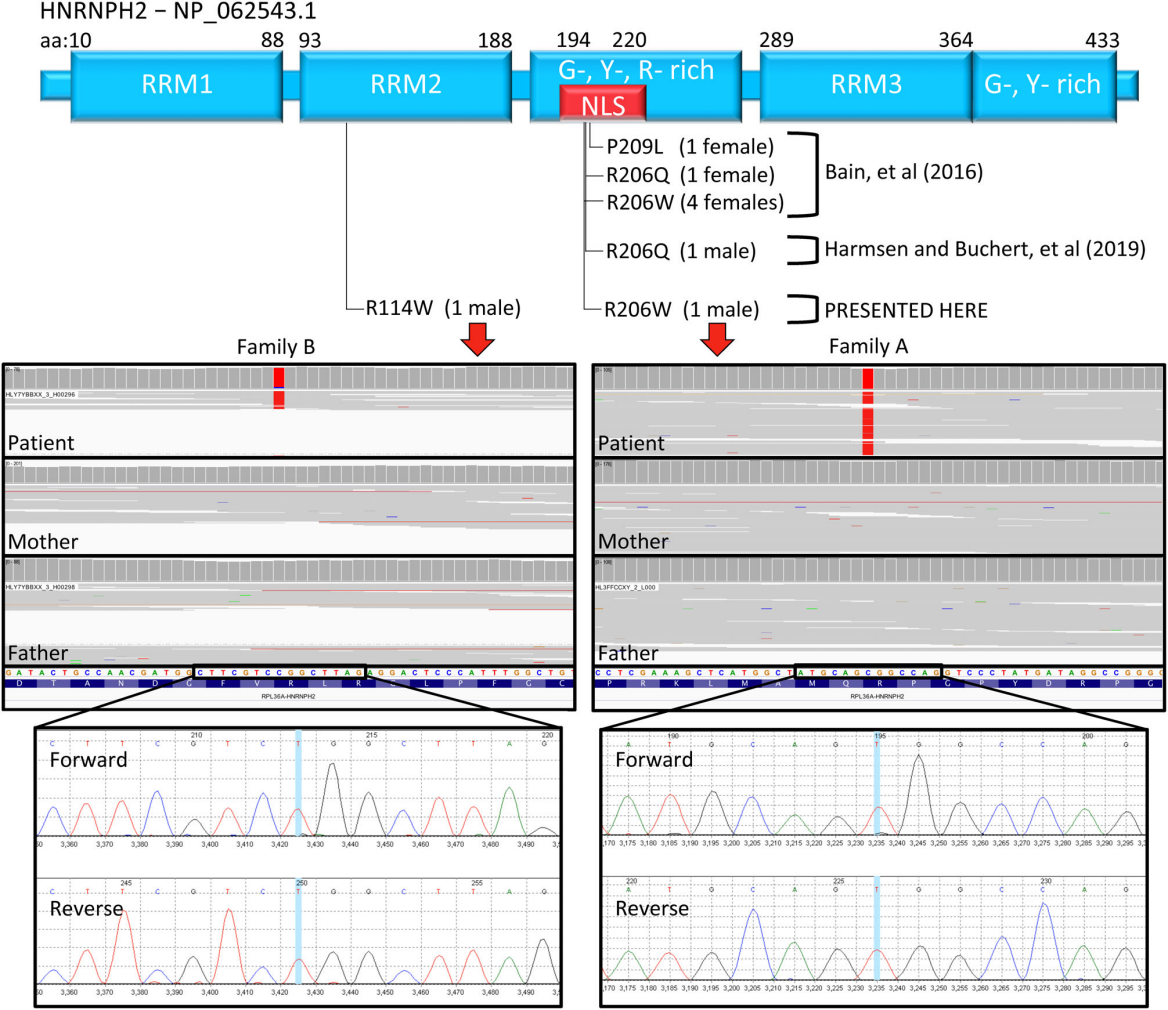
HNRNPH2 is pictured at the top with the location of known causal mutations depicted below. Patient A's mutation (right) is in the nuclear localization sequence, and has been previously described in females by Bain, et al [2], while patient B's mutation (left) is in the second RNA recognition motif (RRM). WES (middle) reveals both patient's mutations are de novo. Mutations were confirmed by Sanger sequencing (bottom)

Patient A is a 5‐year‐old male with global developmental delay. Development was normal until 3 months of age when his parents noted lack of head control and unusual posturing of his extremities. At age 2, a G‐tube was placed and remains his primary nutrition source. MRI at age 3 raised the possibility of a remote, mild hypoxic brain injury, and electromyography showed signs of chronic myopathy. Peripheral nerve disease, Pompe disease, and spinal muscular atrophy were ruled out.

At age 5, he required total support of his head and assistance keeping his mouth closed. He could not sit or crawl but could rotate while lying on his back. He was able to move his hands and fingers, but displayed extreme hypotonia in all extremities and trunk. He had no dysmorphic features. He was non‐verbal but appeared to have some comprehension. He had a happy disposition and was social. He was thought to have a congenital myasthenic syndrome, a congenital myopathy, or a severe, early‐onset mitochondrial disorder.

Whole exome sequencing (WES) was performed on the patient and biological parents revealing a de novo, missense mutation, HNRNPH2(R206W), that has been previously associated with MRXSB.^2^ The variant was confirmed by Sanger sequencing. Phenotypic overlaps with heterozygous females include developmental delay with regression, tone abnormalities, brain abnormalities, and growth problems.

Patient B is an 8‐year‐old male with global developmental delay, microcephaly, failure to thrive, intractable epilepsy, hypotonia, and cortical visual impairment. A vagal nerve stimulator was placed at age 2. At certain times in his life he would have over 10 generalized tonic‐clonic and dozens of myoclonic seizures daily. He was non‐verbal and did not follow commands. There were frequent athetoid movements of the upper extremities. Dyskinetic movements of the face and tongue were observed. Feeding problems required placement of a G‐tube.

WES identified a private, de novo, missense mutation in *HNRNPH2* (c.340C>T, p.Arg114Trp) that was confirmed by Sanger sequencing. The variant has a CADD PHRED of 22.4 and is considered likely pathogenic by the American College of Medical Genetics (ACMG) classification (rules: PS2, PM2^3^). The mutation is predicted to affect protein function by SIFT (http://sift.bii.a-star.edu.sg) with a score of 0.00 (median sequence conservation = 3.07, sequences represented = 29), and MutationTaster (http://mutationtaster.org) predicts it is disease causing (accuracy = 0.9999, converted rank score = 0.5881). WES revealed five reference reads (7%) and 64 variant reads (93%) at the position, implying low level mosaicism for the reference allele. Phenotypic overlaps with MRXSB include developmental delay, seizures, tone abnormalities, and growth problems.

A second variant with an ACMG classification of “uncertain significance” (rules: none meet criteria^3^) was identified in patient B, inherited from his neurologically normal mother, in *GRIA3* (c.419A>G, p.Q140R), encoding a glutamate receptor associated with mental retardation, X‐linked 94. One male is hemizygous for the mutation, and one female is a carrier in gnomAD^4^ (accessed March 2019). The CADD PHRED is 26.3. The mutation is predicted to be tolerated by SIFT with a score of 0.16 (median sequence conservation = 3.15, sequences represented = 26), while MutationTaster predicts it to be disease causing (accuracy = 1, converted rank score = 0.8103). It is worth noting that missense variants within this gene that are associated with mental retardation are located between amino acid 450 and the C‐terminus of the protein—not in the N‐terminus where this mutation was found—and have been associated with macrocephaly,^5^ while *HNRNPH2* has been associated with microcephaly.^1^, ^2^ Furthermore, of ClinVar's 151 known pathogenic variants in *GRIA3*, only five are missense mutations (<4.0%), implying loss‐of‐function of GRIA3 is required for pathogenicity.

Interestingly, the PolyPhen‐2 (http://genetics.bwh.harvard.edu/pph2) prediction algorithm predicts GRIA3(Q140R) as “probably damaging” with a score of 0.999 (sensitivity = 0.14, specificity = 0.99), while the private HNRNPH2(R114W) mutation is predicted “benign” with a score of 0.105 (sensitivity = 0.93, specificity = 0.86). However, the known clinical pathogenic variant carried by patient A, HNRNPH2(R206W) is also predicted by PolyPhen‐2 to be “benign” with a score of 0.028 (sensitivity = 0.95, specificity = 0.81). Therefore, PolyPhen‐2, alone, is not a good predictor for HNRNPH2 pathogenicity, and comparison to the GRIA3(Q140R) score may not be appropriate. Rather, classification via ACMG guidelines, which also accounts for many prediction algorithms, should be considered the final predictor of pathogenicity. Therefore, we do not believe that the causal variant in patient B is in the *GRIA3* gene, but is the private, de novo mutation, HNRNPH2(R114W), although functional studies will be required to conclusively eliminate either variant as contributive to the phenotype.

Our data support those of Harmsen et al by demonstrating that MRXSB mutations in *HNRNPH2* are not embryonically lethal in males, and we add to the literature that other deleterious mutations within the gene are likely capable of producing a range of overlapping phenotypes.

## ETHICS STATEMENT

All participants were consented and enrolled into the Center for Rare Childhood Disorders (C4RCD) research protocol at the Translational Genomics Research Institute (TGen). Written consent for the both children was obtained from the parents. Verbal assent was obtained for participants 7‐17 years of age when appropriate. The study protocol and consent documents were approved by the Western Institutional Review Board (WIRB # 20120789).

2

## Data Availability

The data that support the findings of this study are available on request from the corresponding author. The data are not publicly available due to privacy or ethical restrictions.

## References

[1] Harmsen S , Buchert R , Mayatepek E , Haack TB , Distelmaier F . Bain type of X‐linked syndromic mental retardation in boys. Clin Genet. 2019 10.1111/cge.13524. 30887513

[2] Bain JM , Cho MT , Telegrafi A , et al. Variants in HNRNPH2 on the X chromosome are associated with a neurodevelopmental disorder in females. Am J Hum Genet. 2016;99(3):728‐734. 10.1016/j.ajhg.2016.06.028. 27545675 PMC5011042

[3] Richards S , Aziz N , Bale S , et al. Standards and guidelines for the interpretation of sequence variants: a joint consensus recommendation of the American College of Medical Genetics and Genomics and the Association for Molecular Pathology. Genet Med. 2015 10.1038/gim.2015.30. PMC4544753 25741868

[4] Karczewski KJ , Francioli LC , Tiao G , et al. Variation across 141,456 human exomes and genomes reveals the spectrum of loss‐of‐function intolerance across human protein‐coding genes. bioRxiv. 2019 10.1101/531210.

[5] Wu Y , Arai AC , Rumbaugh G , et al. Mutations in ionotropic AMPA receptor 3 alter channel properties and are associated with moderate cognitive impairment in humans. Proc Natl Acad Sci U S A. 2007;104(46):18163‐18168. 10.1073/pnas.0708699104. 17989220 PMC2084314

